# Durability Improvement of Pt/RGO Catalysts for PEMFC by Low-Temperature Self-Catalyzed Reduction

**DOI:** 10.1186/s11671-015-0963-7

**Published:** 2015-06-10

**Authors:** Kang Gyu Sun, Jin Suk Chung, Seung Hyun Hur

**Affiliations:** School of Chemical Engineering, University of Ulsan, Daehak-ro 93, Nam-gu, Ulsan 680-749 South Korea

**Keywords:** Graphene oxide, Hydrogen bubbling, Durability, Oxygen reduction reaction

## Abstract

Pt/C catalyst used for polymer electrolyte membrane fuel cells (PEMFCs) displays excellent initial performance, but it does not last long because of the lack of durability. In this study, a Pt/reduced graphene oxide (RGO) catalyst was synthesized by the polyol method using ethylene glycol (EG) as the reducing agent, and then low-temperature hydrogen bubbling (LTHB) treatment was introduced to enhance the durability of the Pt/RGO catalyst. The cyclic voltammetry (CV), oxygen reduction reaction (ORR) analysis, and transmittance electron microscopy (TEM) results suggested that the loss of the oxygen functional groups, because of the hydrogen spillover and self-catalyzed dehydration reaction during LTHB, reduced the carbon corrosion and Pt agglomeration and thus enhanced the durability of the electrocatalyst.

## Background

Currently, polymer electrolyte membrane fuel cells (PEMFCs) are considered as one of the most environmentally friendly energy sources and a promising candidate as next-generation power sources for stationary systems and portable applications owing to their low operation temperature, fast start-up, and high energy efficiency and power density [1–3]. The electrochemical corrosion of the carbon supports in PEMFC catalysts is regarded as a main cause of undermining durability when operated for extended periods of time. Thus, the supporting material where catalyst nanoparticles are anchored plays an important role for enhancement of long-term durability. Carbon black (CB) is the most common commercial supporting material for PEMFC catalysts. Despite several advantages such as a large surface area and high conductivity as a porous carbonaceous material, its poor long-term stability is considered as the most critical problem to be solved [4–7]. To improve the long-term durability of PEMFC catalysts, carbon nanotube (CNT) and graphene containing robust graphitic structures have been widely studied as supporting materials [8–16]. Graphene oxide (GO) have abundant surface functional groups and defects, which are chemically active sites for use in catalytic reactions and also act as the anchoring sites for metal nanoparticles, making it a promising supporting material for electrocatalysts for PEMFCs [17]. However, excessive amount of oxygen-containing functional groups can reduce the electrical conductivity and electrochemical stability of CNT and graphene, which can make them weak to chemical oxidation and thus deteriorate the long-term durability of supported electrocatalysts [18].

In this study, low-temperature hydrogen bubbling (LTHB) was conducted to prepare Pt/reduced graphene oxide (RGO) with enhanced durability by the effect of self-catalyzed dehydration of the functional group. LTHB effectively removed the functional groups without the agglomeration of Pt particles. The LTHB-treated Pt/RGO showed highly improved long-term durability compared to the nontreated Pt/RGO after an accelerated durability test for 2 h, because of the less agglomeration of Pt particles. As the LTHB time increased, the long-term durability was improved. The physical and electrochemical characteristics of the fabricated catalysts were examined by thermogravimetric analysis (TGA), X-ray photoelectron spectroscopy (XPS), X-ray diffraction (XRD), transmission electron microscopy (TEM), Raman spectroscopy, cyclic voltammetry (CV), and oxygen reduction reaction (ORR) measurements.

## Methods

### Preparation of Catalysts

GO was prepared by Hummer’s method with expandable graphite (Grade 1721, Asbury Carbon, Co., Ltd., USA) [19]. To prepare Pt/RGO, 50 mg GO was first dispersed in 200 mL ethylene glycol (EG; Daejung Chemicals, Republic of Korea), and then the precalculated amount of metal precursor, H_2_PtCl_6_ · 6H_2_O (Sigma-Aldrich, USA), was added drop by drop to the GO suspension under magnetic stirring. The pH of the suspension was adjusted to 11 using 1 M NaOH solution and was heated and stirred at 110 °C for 90 min to reduce Pt precursors to Pt nanoparticles. The final product was collected by filtration followed by washing with ethanol and DI water. The obtained catalyst was dried in a vacuum oven at 70 °C overnight. The LTHB-treated catalyst (Pt/RGO-R) was prepared by the bubbling of hydrogen gas for 24 h at 80 °C in water [18]. The effects of LTHB time were investigated by using 12, 24, and 36 h of LTHB. The loading amount of Pt was kept ~40 wt% for all the catalysts to set it the same as the commercial Pt/C (40 wt% HiSPEC^TM^ 4000, Johnson Matthey) catalyst, which was analyzed by TGA (TA Instrument TGA Q50, USA).

### Instrumental Analysis

The chemical compositions and functional groups of GO were examined by X-ray photoelectron spectroscopy (XPS; Thermo Fisher K-alpha, UK) and element analysis (EA; Thermo Scientific Flash 2000, Netherlands). Raman analysis was conducted to identify the change in the defects in the GO using a DXR Raman microscope (Thermo Scientific, USA) with incident light at a wavelength of 532 nm. XRD (Rigaku RAD-3C, Japan) analysis was performed to investigate the crystal structure and size of Pt particles in each catalyst. XRD data were collected in the range of 10° to 100° with Cu Kα radiation (*λ* = 1.5418 Å) at 40 kV and 30 mA. TGA (TA Instrument TGA Q50) was used to determine the loading amount of Pt on GO at a heating rate of 10 °C min^−1^ ranging from room temperature up to 800 °C. The morphology and distribution of Pt particles on GO were characterized by TEM (JEOL JEM-2100 F, Japan).

### Electrochemical Analysis

Cyclic voltammetry (CV; BioLogic, SP-50, USA) was measured using a half-cell system with three electrodes for electrochemical analysis. A Pt wire and an Ag/AgCl electrode were used as counter and reference electrodes, respectively. A glassy carbon electrode (GCE; 3 mm in diameter) was polished and used as the working electrode after 3 μL of catalyst ink was coated on it. The uniform catalyst ink was synthesized by mixing IPA with 5 wt% Nafion (Sigma-Aldrich, USA) with 5 mg catalyst followed by 30 min ultrasonic treatment. CV measurements were performed from −0.2 to 1.0 V to measure the electrochemical surface area (ECSA) of catalysts in nitrogen-saturated 0.5 M H_2_SO_4_ at a scan rate of 50 mV s^−1^, and the durability of the catalysts was investigated by repeating 200 cycles between 0.4 and 1.2 V at the same scan rate. Linear sweep voltammetry (LSV) for oxygen reduction reaction (ORR) activity was measured using a rotating disk electrode (RDE; 3 mm in diameter) in an oxygen-saturated 0.5 M H_2_SO_4_ electrolyte between 0.9 and 0 V at a scan rate of 10 mV s^−1^, and the ORR durability was also examined at a rotating rate of 1600 rpm for 200 cycles. All the experiments were carried out at 25 °C. Moreover, electrochemical impedance spectroscopy (EIS) was performed in the frequency range of 0.01 Hz to 100 kHz.

## Results and Discussion

**Fig. 1:**
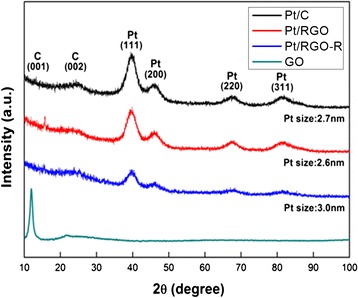
XRD patterns of Pt/C, Pt/RGO, Pt/RGO-R, and GO

The XRD patterns shown in Fig. 1 exhibit representative diffraction peaks at 39.8°, 46.3°, 68.2°, and 81.6°, corresponding to the (111), (200), (220), and (311) planes of the face-centered cubic (FCC) structure of Pt (JCPDS #04-0802), respectively.

The Pt particle size in Pt/RGO was ~2.6 nm, which was similar to that of the commercial Pt/C catalyst (2.7 nm), indicating uniform distribution of Pt nanoparticles on GO even at a high loading amount. After the LTHB treatment, there was a slight increase in the particle size (3.0 nm), which can be because of the redeposition of the dissociated Pt ions from the Pt particles during LTHB [20]. The XRD peak at ~11° of GO was attributed to the extended sheet-to-sheet distance by the large amount of functional groups and captured water molecules between hydrophilic sheets, which shifted to around 23° in Pt/RGO, because of the decrease in the interlayer distance of GO by the reduction of functional groups during Pt deposition [21]. A slight shift of the carbon (002) peak of Pt/RGO-R was ascribed to the further reduction of functional groups during the LTHB treatment.

**Fig. 2:**
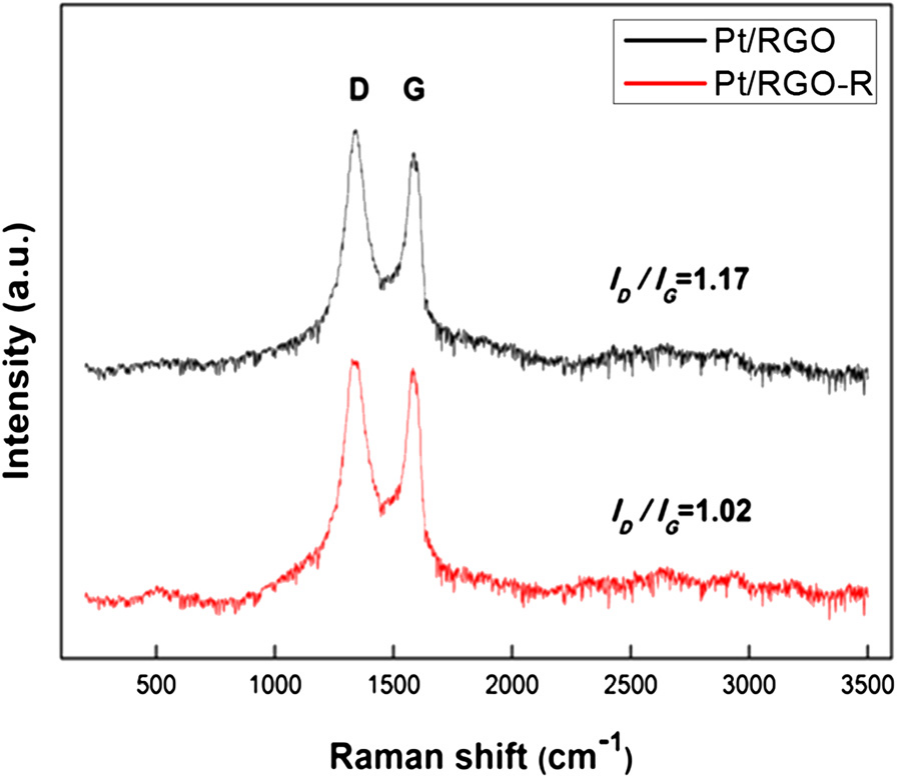
Raman spectra of Pt/RGO and Pt/RGO-R

The reduction of the functional group in Pt/RGO by the LTHB treatment was confirmed by the Raman spectrum as shown in Fig. 2. Pt/RGO and Pt/RGO-R exhibit the G and D bands at similar positions, ~1589 and 1334 cm^−1^, respectively. The G band originated from a vibration mode of adjacent carbon atoms in the hexagonal structure moving in opposite directions and has an E2g symmetry. The D band exhibits crystal defects formed during the exfoliation of graphite [22–24]. After the LTHB treatment, the *I*_D_/*I*_G_ value decreased from 1.17 (Pt/RGO) to 1.02 (Pt/RGO-R), indicating the decrease in defects by the reduction of the functional groups and restoration of the C = C bonds.

**Fig. 3:**
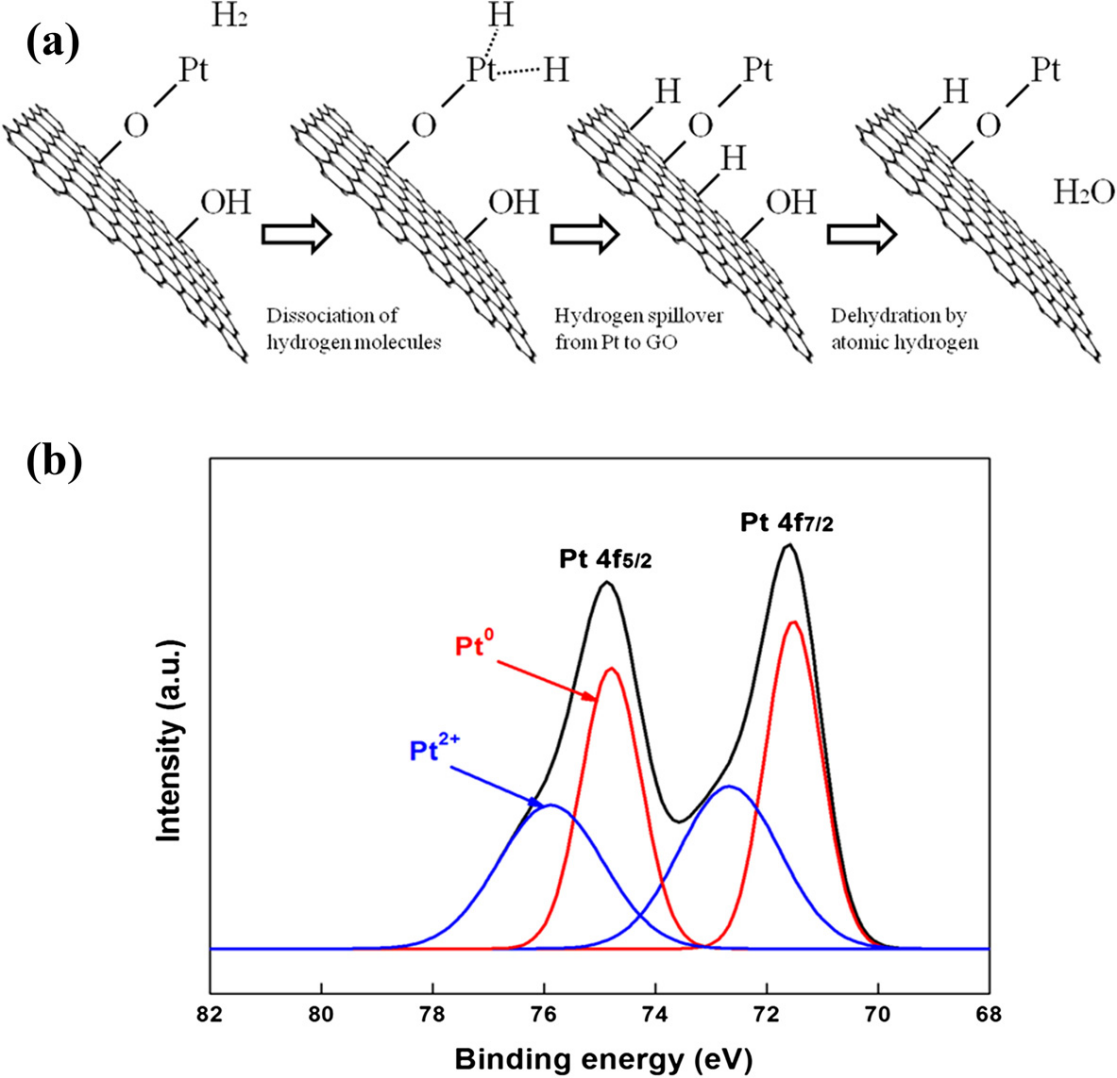
**a** Schematic representation of self-catalyzed dehydration on Pt/RGO. **b** Deconvoluted Pt 4f XPS spectra of the Pt/RGO-R catalyst

The schematic representation of self-catalyzed dehydration of Pt/RGO during the LTHB treatment is shown in Fig. 3a. First, hydrogen molecules dissociate into atomic hydrogens by Pt nanoparticles, which migrate from Pt to functional groups such as the hydroxyl group (–OH) and epoxy group (–O–), followed by dehydration forming H_2_O on the GO surface [18, 25]. The formation of Pt–O–C bonds can be confirmed by the existence of the Pt^2+^ XPS peak as shown in Fig. 3b [26].

**Fig. 4:**
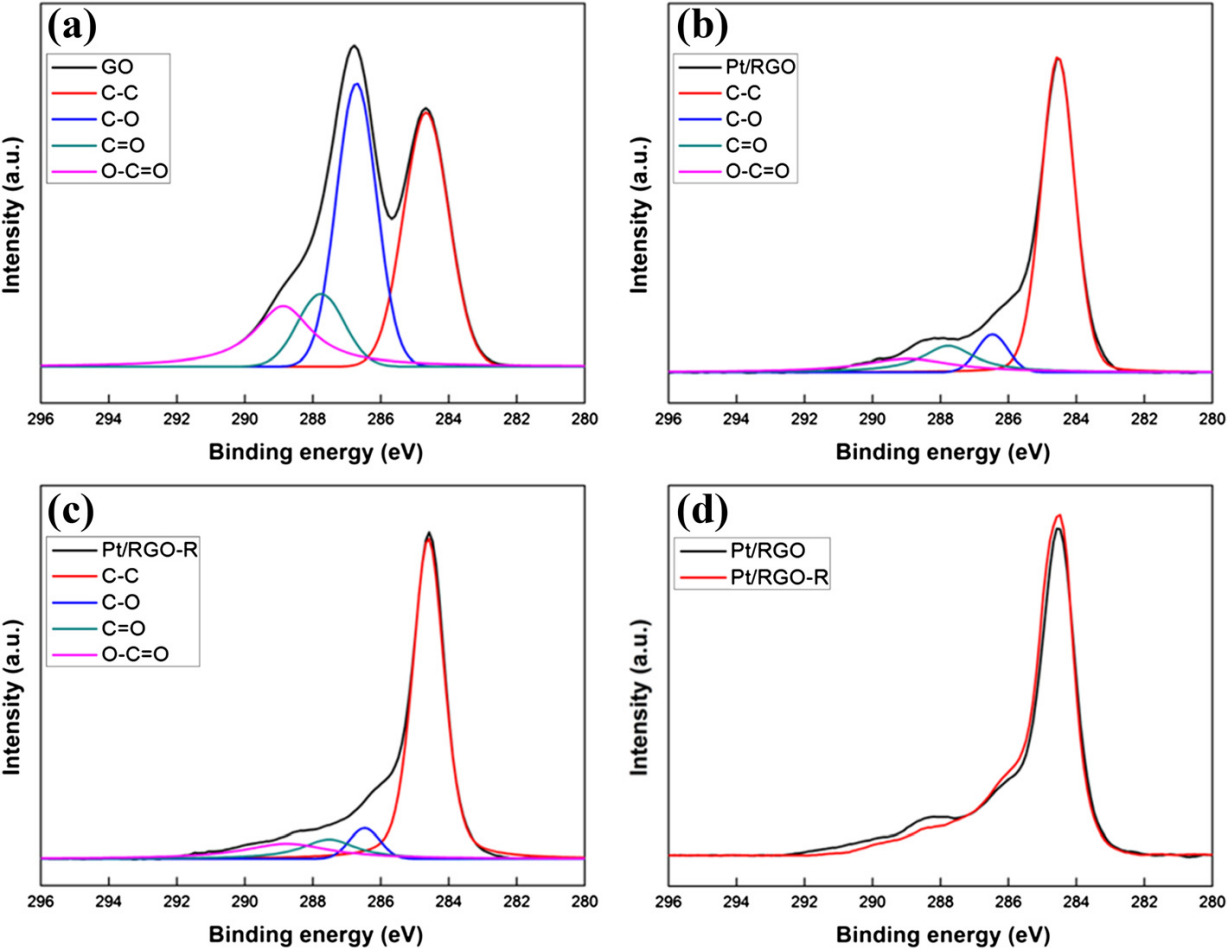
XPS C1s spectra of **a** GO, **b** Pt/RGO, and **c** Pt/RGO-R. **d** Comparison of C1s spectra between Pt/RGO and Pt/RGO-R

The reduction of the functional groups during the LTHB treatment was further confirmed by XPS and EA. As shown in Fig. 4, the C1s XPS spectrum of the pristine GO exhibits four different peaks centered at 284.5, 286.5, 287.7, and 288.9 eV, corresponding to C–C, C–O, C = O, and O–C = O groups in GO, respectively [27]. Pt/RGO shows highly reduced functional groups over the pristine GO, indicating the co-reduction of Pt and GO during the Pt loading step [28]. Moreover, a further decrease in the C–O and C = O groups and increase in the C–C bond intensities were observed after the LTHB treatment by the self-catalyzed dehydration. EA results summarized in Table 1 exhibit highly decreased O/C ratio of Pt/RGO-R over Pt/RGO, which also confirms the reduction of oxygen-containing functional groups during the LTHB treatment.

**Table 1:** Element analysis of GO, Pt/RGO, and Pt/RGO-R

Sample	Atomic content (%)			
	C	O	Others	O/C ratio
GO	48.7	44.9	3.5	0.92
Pt/RGO	55.9	10.8	2.4	0.19
Pt/RGO-R	56.4	2.1	1.5	0.04

**Fig. 5:**
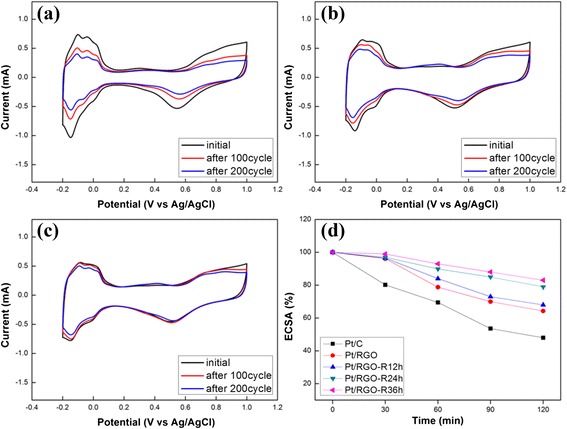
CV of **a** Pt/C, **b** Pt/RGO, and **c** Pt/RGO-R in N_2_-saturated 0.5 M H_2_SO_4_ solution at a scan rate of 50 mV s^−1^ for 200 cycles and **d** comparison of normalized ECSA as a function of LTHB treatment time

The changes in the ECSA for various catalysts after repeated electrochemical cycles were measured using a CV instrument. As shown in Fig. 5 and Table 2, Pt/RGO exhibits better stability than Pt/C. Moreover, the stability of Pt/RGO-R further improved after the LTHB treatment. Pt/C showed as much as 52.1 % decrease in the ESCA after 200 repeated cycles, but those of Pt/RGO and Pt/RGO-R dropped only 35.7 and 21 %, respectively, which clearly indicate improved long-term stability without sacrificing the initial ECSA after the LTHB treatment. The effect of the LTHB treatment time was evaluated and is shown in Fig. 5d. The stability of Pt/RGO only slightly increased after 12 h of treatment; however, after another 12 h, a noticeable increase in the stability was observed. There was no further distinct improvement after another 12 h of treatment.

**Table 2:** ECSA change of each catalyst during repeated CV cycles in a half-cell system

Sample	ECSA (m^2^ g^−1^)		
	Initial	After	
		100 cycles	200 cycles
Pt/C	54.1	37.6	25.9
(deactivation)	(−30.5 %)		(−52.1 %)
Pt/RGO	34.5	27.2	22.2
(deactivation)	(−21.2 %)		(−35.7 %)
Pt/RGO-R	34.8	31.3	27.5
(deactivation)	(−10.1 %)		(−21.0 %)

**Fig. 6:**
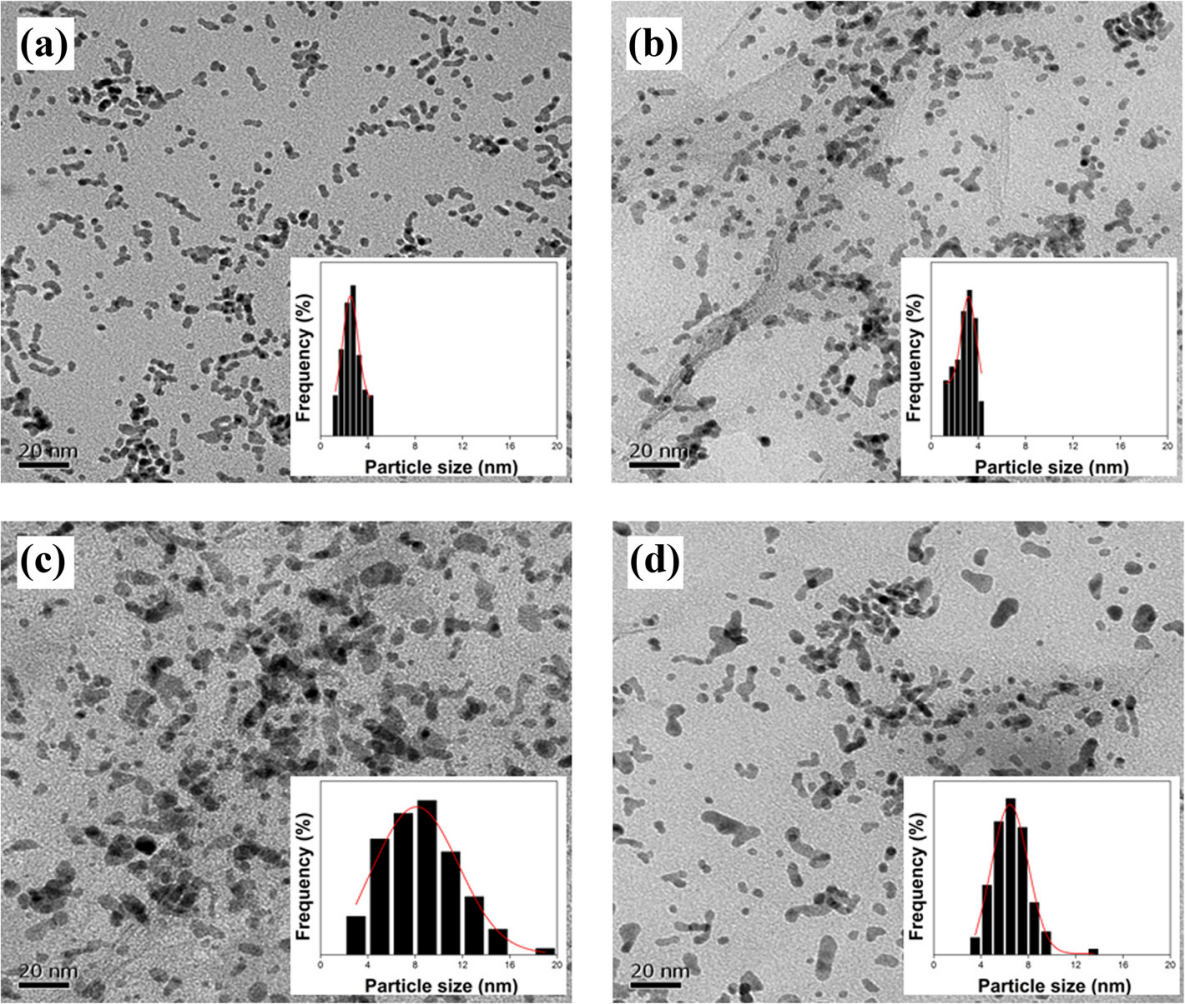
TEM images of initial **a** Pt/RGO and **b** Pt/RGO-R and final **c** Pt/RGO and **d** Pt/RGO-R after repeated CV cycles. *Insets* are the histograms of Pt size distributions

**Fig. 7:**
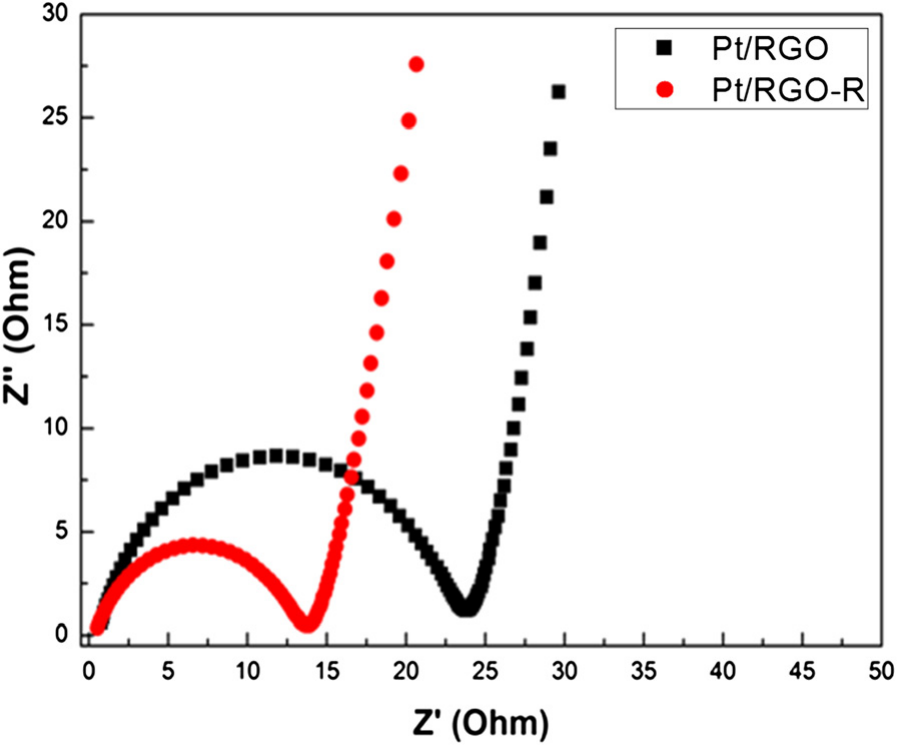
The Nyquist plots of Pt/RGO and Pt/RGO-R at a frequency range from 0.01 Hz to 100 kHz

The agglomeration of the Pt nanoparticles after repeated CV cycles was analyzed by TEM images as shown in Fig. 6. Initially, Pt nanoparticles are uniformly dispersed over the GO surface. After 200 repeated CV cycles, a high level of agglomeration (224 %) was observed in the nontreated Pt/RGO (Table 3). In contrast, only 106 % of agglomerate for Pt/RGO-R was observed, which clearly indicates that LTHB improves the long-term stability of the Pt/RGO catalyst. Because of the better graphitic structure and less functional groups in GO after the LTHB treatment, GO became more resistant to the chemical corrosion during repeated cycles, which resulted in the less agglomeration of Pt nanoparticles on GO-R than that on pristine GO [29]. The Nyquist plots of Pt/RGO and Pt/RGO-R measured by electrochemical impedance spectroscopy (EIS) are shown in Fig. 7. The smaller diameter of the semicircle of Pt/RGO-R than that of Pt/RGO indicates a low resistance of Pt/RGO-R, which also suggests the recovered sp^2^ networks after LTHB [30].

**Table 3:** Mean particle size and agglomeration ratio of Pt/RGO and Pt/RGO-R

Sample	TEM		
	Pt particle size (nm)		Agglomeration ratio (%)
	Initial	After	
Pt/RGO	2.5	8.1	+224
Pt/RGO-R	3.1	6.4	+106

**Fig. 8:**
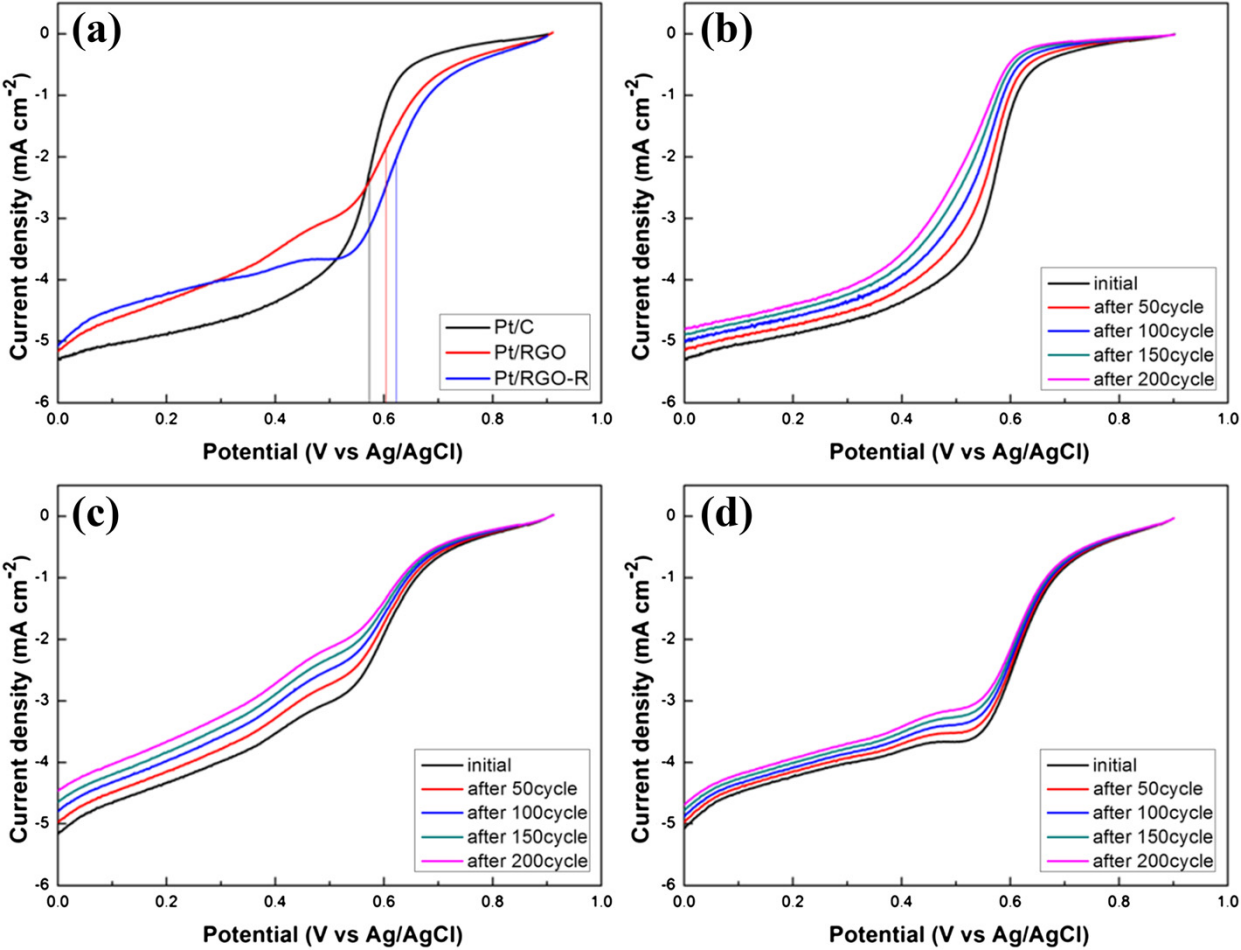
**a** Initial ORR polarization curves of Pt/C, Pt/RGO, and Pt/RGO-R. Changes of ORR polarization curves of **b** Pt/C, **c** Pt/RGO, and **d** Pt/RGO-R in O_2_-saturated 0.5 M H_2_SO_4_ solution at a scan rate of 10 mV s^–1^ for 200 cycles

As shown in Fig. 8a, the half-wave potential (*E*½) corresponding to one half of the diffusion current was used to evaluate the ORR activity of the catalysts. Pt/C and Pt/RGO showed an *E*½ of about ~47.4 and 12.3 mV, respectively, which shifted from that of Pt/RGO-R, indicating better ORR activity of Pt/RGO-R because of the enhanced charge transfer between Pt and GO that originated from the increased electrical conductivity of GO-R after the LTHB treatment. The deactivation of the ORR activities of Pt/C, Pt/RGO, and Pt/RGO-R was measured by conducting 200 repeated cycles. As shown in Fig. 8, Pt/RGO-R exhibited a less decrease of *E*½ than those of Pt/C and Pt/RGO. The *E*½ shifts of Pt/C, Pt/RGO, and Pt/RGO-R after 200 repeated ORR cycles were 53.4, 19, and 4.7 mV, respectively, indicating that the self-catalyzed reduction by hydrogen minimizes carbon corrosion, and thus Pt agglomeration, leading to highly improved long-term durability.

## Conclusions

In this study, a highly durable Pt/RGO-R electrocatalyst was prepared by LTHB treatment. The reduction of the functional groups and restoration of sp^2^ networks in GO were confirmed by the XPS and Raman spectra. Both the CV and ORR tests demonstrate that Pt/RGO-R showed a better long-term durability than the nontreated Pt/RGO and Pt/C. Pt/RGO-R exhibited only 21 % drop in ECSA after 200 repeated CV cycles, which is approximately 1.7 times higher than that of the nontreated Pt/RGO and 2.5 times higher than that of the commercial Pt/C catalyst. The TEM results show that the agglomeration of Pt nanoparticles after repeated CV cycles was highly suppressed by the self-catalyzed dehydration, resulting in less carbon support corrosion and thus improved long-term durability.
